# P-glycoprotein attenuates DNA repair activity in multidrug-resistant cells by acting through the Cbp-Csk-Src cascade

**DOI:** 10.18632/oncotarget.15065

**Published:** 2017-02-03

**Authors:** Li-Fang Lin, Ming-Hsi Wu, Vijaya Kumar Pidugu, I-Ching Ho, Tsann-Long Su, Te-Chang Lee

**Affiliations:** ^1^ Institute of Biomedical Sciences, Academia Sinica, Taipei 11529, Taiwan; ^2^ Taiwan International Graduate Program in Molecular Medicine, National Yang-Ming University, Academia Sinica, Taipei 11529, Taiwan; ^3^ Institute of Pharmacology, National Yang-Ming University, Taipei 11221, Taiwan

**Keywords:** P-glycoprotein, multidrug resistance, Cbp-Csk-Src cascade, DNA repair proteins

## Abstract

Recent studies have demonstrated that P-glycoprotein (P-gp) expression impairs DNA interstrand cross-linking agent-induced DNA repair efficiency in multidrug-resistant (MDR) cells. To date, the detailed molecular mechanisms underlying how P-gp interferes with Src activation and subsequent DNA repair activity remain unclear. In this study, we determined that the C-terminal Src kinase-binding protein (Cbp) signaling pathway involved in the negative control of Src activation is enhanced in MDR cells. We also demonstrated that cells that ectopically express P-gp exhibit reduced activation of DNA damage response regulators, such as ATM, Chk2, Braca1 and Nbs1 and hence attenuated DNA double-strand break repair capacity and become more susceptible than vector control cells to DNA interstrand cross-linking (ICL) agents. Moreover, we demonstrated that P-gp can not only interact with Cbp and Src but also enhance the formation of inhibitory C-terminal Src kinase (Csk)-Cbp complexes that reduce phosphorylation of the Src activation residue Y416 and increase phosphorylation of the Src negative regulatory residue Y527. Notably, suppression of Cbp expression in MDR cells restores cisplatin-induced Src activation, improves DNA repair capacity, and increases resistance to ICL agents. Ectopic expression of Cbp attenuates cisplatin-induced Src activation and increases the susceptibility of cells to ICL agents. Together, the current results indicate that P-gp inhibits DNA repair activity by modulating Src activation via Cbp-Csk-Src cascade. These results suggest that DNA ICL agents are likely to have therapeutic potential against MDR cells with P-gp-overexpression.

## INTRODUCTION

Multidrug resistance (MDR) is a significant obstacle to the success of chemotherapy in cancer patients [1]. Although MDR may be attributed to various mechanisms, it is often associated with increased expression of ATP-binding cassette (ABC) transporter family members, which extrude anticancer drugs out of cells [2, 3]. The *MDR1* gene product, P-glycoprotein (P-gp), is one of the most well-known ABC transporters. ABC transporters expel a broad range of bioactive chemicals [4], including various anticancer drugs, such as vinblastine, vincristine, doxorubicin and paclitaxel [5, 6]. Thus, overexpression of P-gp in tumor tissues is a prognostic indicator associated with poor response to chemotherapy and poor clinical outcome [7–9]. Numerous agents have been identified or developed to modify, modulate, or reverse the P-gp-mediated MDR phenotype [1, 10, 11]. However, most of those agents were terminated during clinical trials because of their toxicities or unexpected outcomes [12]. Therefore, developing novel agents against P-gp and targeting alternative mechanisms that sensitize MDR cells to therapeutic agents may represent new paths toward overcoming MDR [11, 13].

Alternatively, numerous studies have shown that cancer cells with acquired MDR or ectopically expressed P-gp have increased sensitivity to DNA-damaging agents, including cisplatin [14, 15]. Our previous study has also found that P-gp overexpression attenuates DNA repair in MDR cells damaged by DNA interstrand cross-linking (ICL) agents [16]. However, studies investigating how P-gp interferes with DNA repair are limited. We have previously revealed that Src activation by DNA-damaging agents is significantly reduced by P-gp overexpression in MDR cells [16]. Because Src signaling plays crucial roles in the regulation of the DNA damage response (DDR) [17], our study suggests that P-gp interferes with Src activation.

*Src*, the first identified oncogene encoding a non-receptor tyrosine kinase, plays pivotal roles in coordinating diverse cellular responses involved in differentiation, adhesion and migration [18]. Src is overexpressed and activated in various human cancers [19], suggesting its role in tumor progression. Src also participates in controlling several parameters of cancer metastasis [20] and drug resistance [21, 22]. Several reports have identified associations between Src and resistance to irradiation, cisplatin, and paclitaxel [17, 23, 24]. Src activation-induced drug resistance is likely because of the activation of DNA-PK and enhancement of DNA double-strand break repair [25–27]. In addition, Src causes the dissociation of cyclin-dependent kinase 2 from cyclin A and induces S-phase arrest, thereby enhancing the repair of etoposide-induced DNA damage [28]. Therefore, targeting Src offers a novel therapeutic intervention strategy against cancer, particularly in the augmentation of chemosensitivity [29].

Oncogenic activation of Src requires phosphorylation at tyrosine 416 (pSrc^Y416^) of the catalytic domain, whereas the enzymatic activity of Src is blocked when the tyrosine 527 of the C-terminal regulatory element is phosphorylated (pSrc^Y527^) [30]. Phosphorylation of the C-terminal regulatory Y527 on Src is catalyzed by C-terminal Src kinase (Csk), a unique regulatory tyrosine kinase of the Src family [31]. Src is anchored to specific lipid-raft membrane domains via N-termini, whereas Csk, a cytoplasmic protein, is recruited via adaptor proteins to the membrane and inhibits Src [32]. Among several Csk-binding proteins [33–35], Csk-binding protein (Cbp), also known as phosphoprotein associated with glycosphingolipid-enriched membrane, is exclusively localized to lipid rafts and recruits Csk to efficiently inactivate Src [36, 37]. Therefore, Cbp is known as a transmembrane adaptor protein of Csk. Previous studies have proposed that Cbp acts as a suppressor of Src-mediated cell migration [38], tissue repair [39], and tumor progression [40]. A recent study has also shown that P-gp plays a special role in Cbp recruitment [41]. Accordingly, we hypothesized that Cbp is a critical component involved in the P-gp-mediated regulation of Src in DNA-damaging agent-induced DNA repair. In this study, we conducted experiments to investigate whether P-gp mediates Cbp-Csk-dependent signaling to attenuate Src activation, thereby reducing DNA repair activity in MDR cells.

## RESULTS

### Ectopic expression of P-glycoprotein inhibits cisplatin-induced activation of Src at Y416

**Figure 1:**
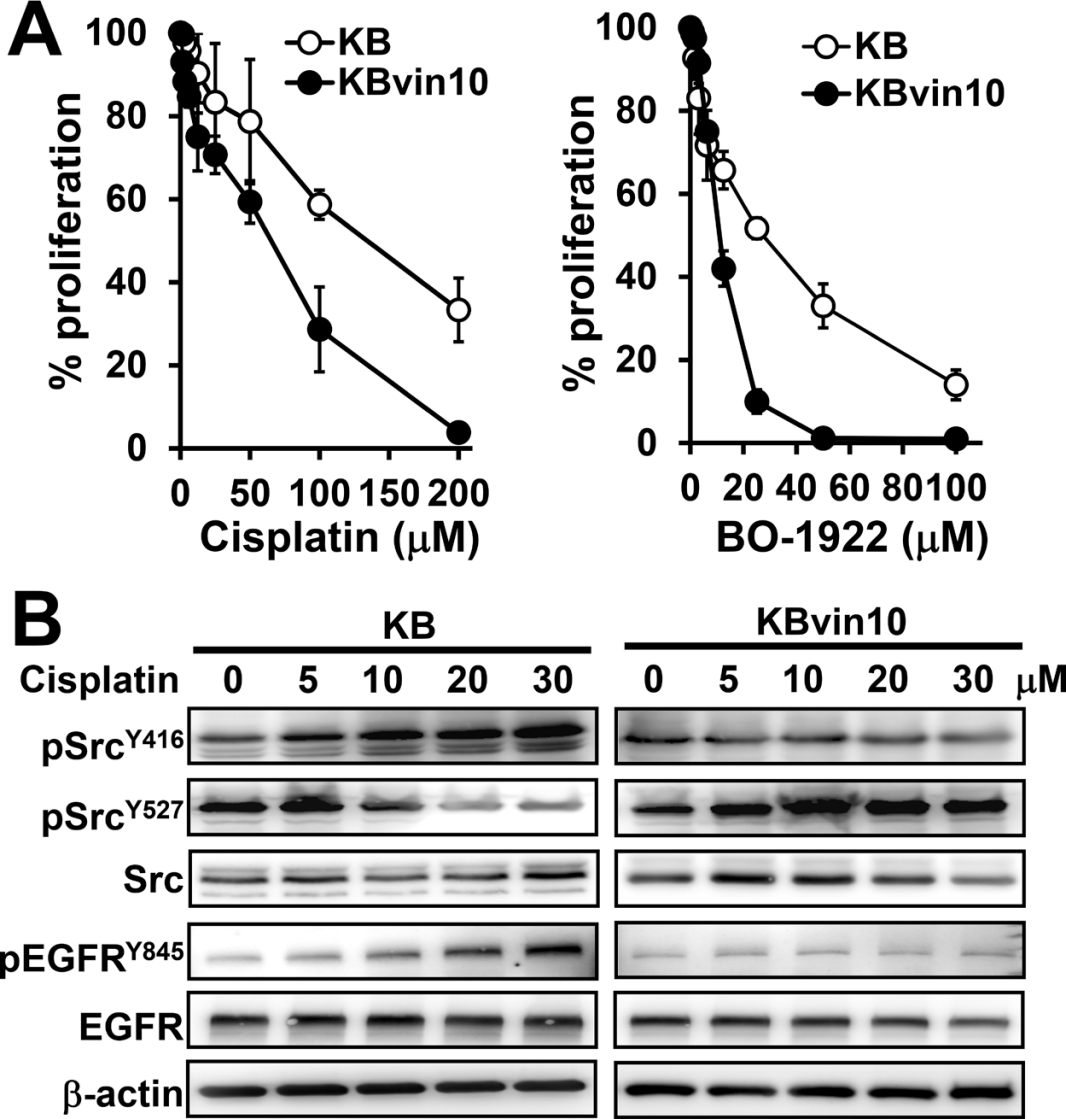
Increased susceptibility to DNA damaging agents and decreased Src activation in P-gp overexpressed KBvin10 cells (**A**) Increasing cytotoxic effects of DNA damaging agents to KBvin10 cells. KB and KBvin10 cells were treated with various concentrations of cisplatin or BO-1922 for 1 h, washed with phosphate buffered saline (PBS) and then incubated with fresh medium for 72 h. The cell proliferation was analyzed using Presto-Blue. Bars are SD of three independent experiments. (**B**) No significant Src activation in KBvin10 cells treated with cisplatin. KB or KBvin10 cells were treated with various concentrations of cisplatin for 1 h, washed with PBS, and incubated with fresh medium for 2 h. The levels of Src, pSrc^Y416^ (active) and pSrc^Y527^ (inactive), EGFR and pEGFR^Y845^ were determined by western blotting. β-actin was included as a loading control.

While we previously demonstrated that DNA damage significantly induces Src activation in parental KB cells but not in P-gp-overexpressing KBvin10 cells [16], we first confirmed that KBvin10 cells were more susceptible than KB cells to DNA cross-linking agents, such as cisplatin and BO-1922, a synthesized DNA ICL derivative of indolizino[6,7-*b*]indole (compound 18a in our previous report) [42] (Figure 1A). The IC_50_ values of KB cells to cisplatin and BO-1922 were 3.0 and 1.6 folds higher than those of KBvin10 cells, respectively. We further observed that cisplatin treatment markedly enhanced activated Src (pSrc^Y416^) but significantly reduced inactivated Src (pSrc^Y527^) in KB cells (Figure 1B). On the contrary, pSrc^Y527^ was enhanced whereas pSrc^Y416^ remained at a relatively low level in cisplatin-treated KBvin10 cells (Figure 1B). Moreover, we observed significantly increased EGFR phosphorylation at residue Y^845^ (pEGFR^Y845^), known as Src kinase specific phosphorylation site [43], in KB cells but not in KBvin10 cells treated with cisplatin (Figure 1B). Total EGFR expression was not changed in either KB or KBvin10 cells after cisplatin treatment (Figure 1B), implying that Src activation in cisplatin treated KBvin10 cells was attenuated by overexpressed P-gp.

**Figure 2:**
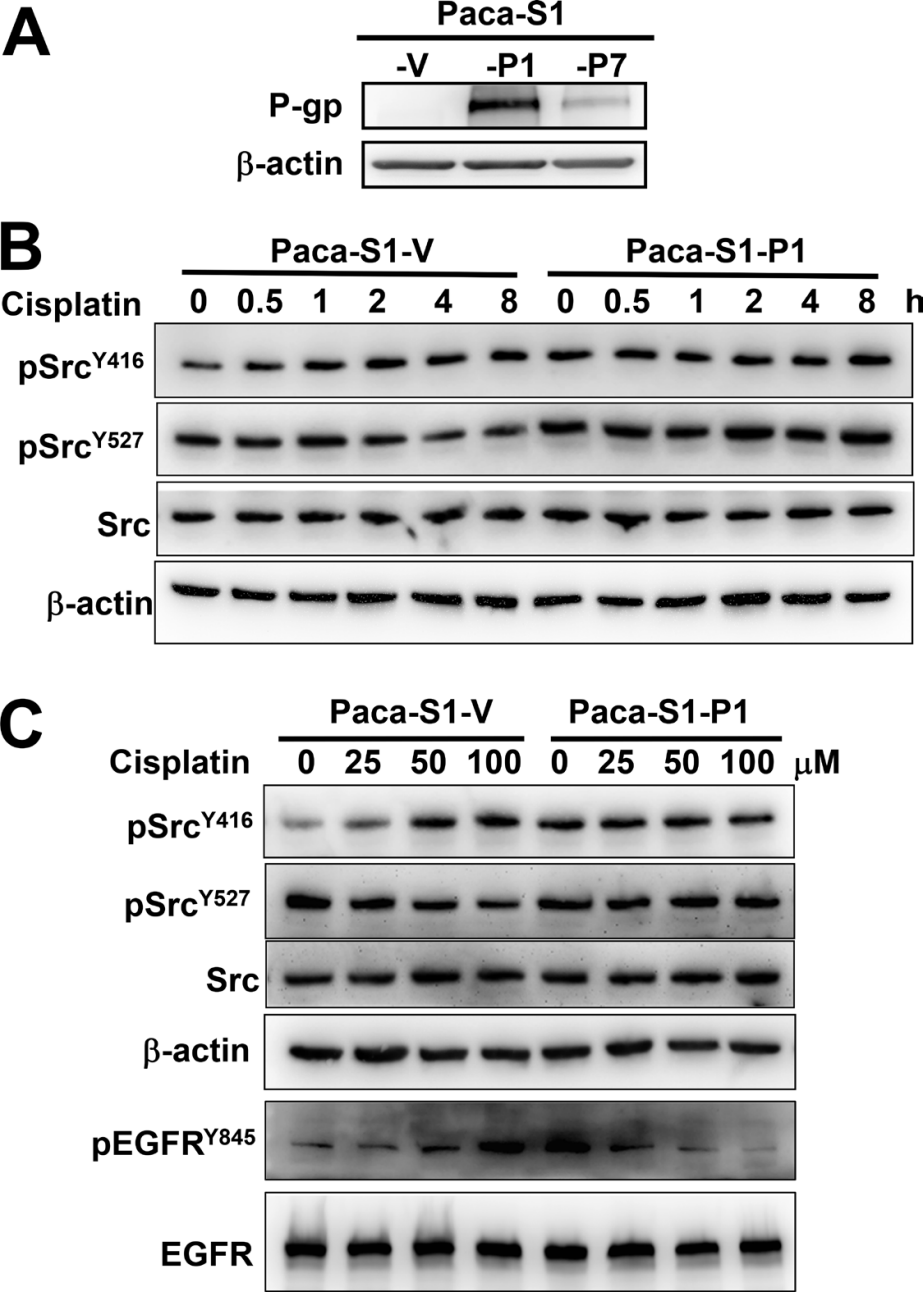
Attenuation of cisplatin-induced Src activation in P-gp overexpressing Paca-S1 cells (**A**) Enhanced expression of P-gp in Paca-S1 cells transfected with a P-gp-expressing vector. Two stable P-gp expressing cell lines, Paca-S1-P1 and -P7, were established and maintained in medium containing 10 nM vincristine. Paca-S1-V cells were transfected with expression vector and served as a control. The protein levels of P-gp were analyzed by western blotting. (**B** and **C**) Time- and dose-dependent modulation of pSrc^Y416^ and pSrc^Y527^ in Paca-S1-V and -P1 cells, respectively. As described in Figure 1, Paca-S1-V and Paca-S1-P1 cells were treated either with 50 μM cisplatin for 1 h, washed, and incubated in the fresh medium for various time periods as indicated (B) or various concentrations for 1 h, washed, and incubated for 4 h (C). The protein levels of total Src, pSrc^Y416^, pSrc^Y527^, EGFR, and pEGFR^Y845^ were analyzed by western blotting.

MDR cells are generally established by long-term drug selection [44]. To further understand how P-gp attenuates DNA-damaging agent-induced Src activation, we ectopically expressed P-gp in Paca-S1 cells and established stable clones (Paca-S1-P1 and Paca-S1-P7) by culturing them in the medium containing vincristine. Because Paca-S1-P1 cells express higher levels of P-gp than Paca-S1-P7 cells (Figure 2A), they were chosen for this study. We first assessed the acquired resistance of Paca-S1-P1 cells to MDR drugs, such as vincristine, vinblastine, doxorubicin, etoposide, and paclitaxel. As summarized in Table 1, Paca-S1-P1 cells were cross-resistant to all of these MDR drugs. However, Paca-S1-P1 cells were more susceptible than Paca-S1-V cells to DNA cross-linking agents, such as cisplatin, melphalan, carboplatin, and BO-1922. These results were consistent to our previous findings [16]. Furthermore, we showed that treatment of Paca-S1-V cells with 50 μM cisplatin for 1 h and followed by incubation in drug-free medium resulted in increased pSrc^Y416^ but decreased pSrc^Y527^ in a time-dependent manner (Figure 2B). The relative intensity of pSrc^Y416^ and pSrc^Y527^ at 8 h was 1.85 ± 0.03 (*n* = 3) and 0.66 ± 0.01 (*n* = 3) in Paca-S1-V cells, respectively. However, no notable change was observed in Paca-S1-P1 cells treated with cisplatin. We further confirmed these findings by treatment of Paca-S1-V cells or Paca-S1-P1 cells with various concentrations of cisplatin for 4 h. As shown in Figure 2C, activated pSrc^Y416^ was increased whereas inactivated pSrc^Y527^ decreased in a dose-dependent manner in Paca-S1-V cells but not in Paca-S1-P1 cells. The relative intensity of pSrc^Y416^ and pSrc^Y527^ at 100 μM to control was 2.27 ± 0.04 (*n* = 4) and 0.53 ± 0.04 (*n* = 4) in Paca-S1-V cells, respectively. However, there was no change in Paca-S1-P1 cells. In addition, we also observed that cisplatin treatment resulted in dose-dependent increase of pEGFR^Y845^ in Paca-S1-V cells but dose-dependent decrease in Paca-S1-P cells. Since KBvin10 and Paca-S1-P1 cells were acquired by selection in medium containing vincristine, we performed similar experiments using KB cells that were transiently expressed P-gp without drug selection. As shown in Supplementary Figure 1, similar results were observed, suggesting that P-gp indeed played certain role on attenuating the Src activation. These results similar to those observed in KBvin10 cells further implicated that P-gp may contribute to the resistance of MDR drugs by attenuation of DNA damaging agent induced Src activation.

**Table 1:** The IC_50_ values of various drugs against Paca-S1-V and P-gp-overexpressing Paca-S1-P1 cells^a^

Drugs	Paca-S1-V	Paca-S1-P1	RF^b^
Vincristine (nM)	3.4 ± 1.2	37,270 ± 317	10,961
Vinblastine (nM)	1.6 ± 0.0	317.2 ± 4.5	198
Doxorubicin (nM)	20.2 ± 6.5	1,389 ± 30.1	69
Etoposide (μM)	2.9 ± 0.3	35.4 ± 4.3	12
Paclitaxel (μM)	0.03 ± 0.00	2.0 ± 0.2	67
Cisplatin (μM)	19.6 ± 0.4	12.6 ± 2.8	0.64
Carboplatin (μM)	56.1 ± 1.8	51.4 ± 0.5	0.91
Melphalan (μM)	109.6 ± 4.0	66.5 ± 5.4	0.61
BO-1922 (μM)	0.52 ± 0.02	0.26 ± 0.08	0.50

^a^IC_50_ values are the drug concentrations that inhibit cell growth by 50%. The data are expressed as means ± SE of three independent experiments.

^b^RF, resistant factor (IC_50_ of Paca-S1-P1 cells/IC_50_ of Paca-S1-V cells).

### Ectopic expression of P-glycoprotein suppresses DNA damage response

**Figure 3:**
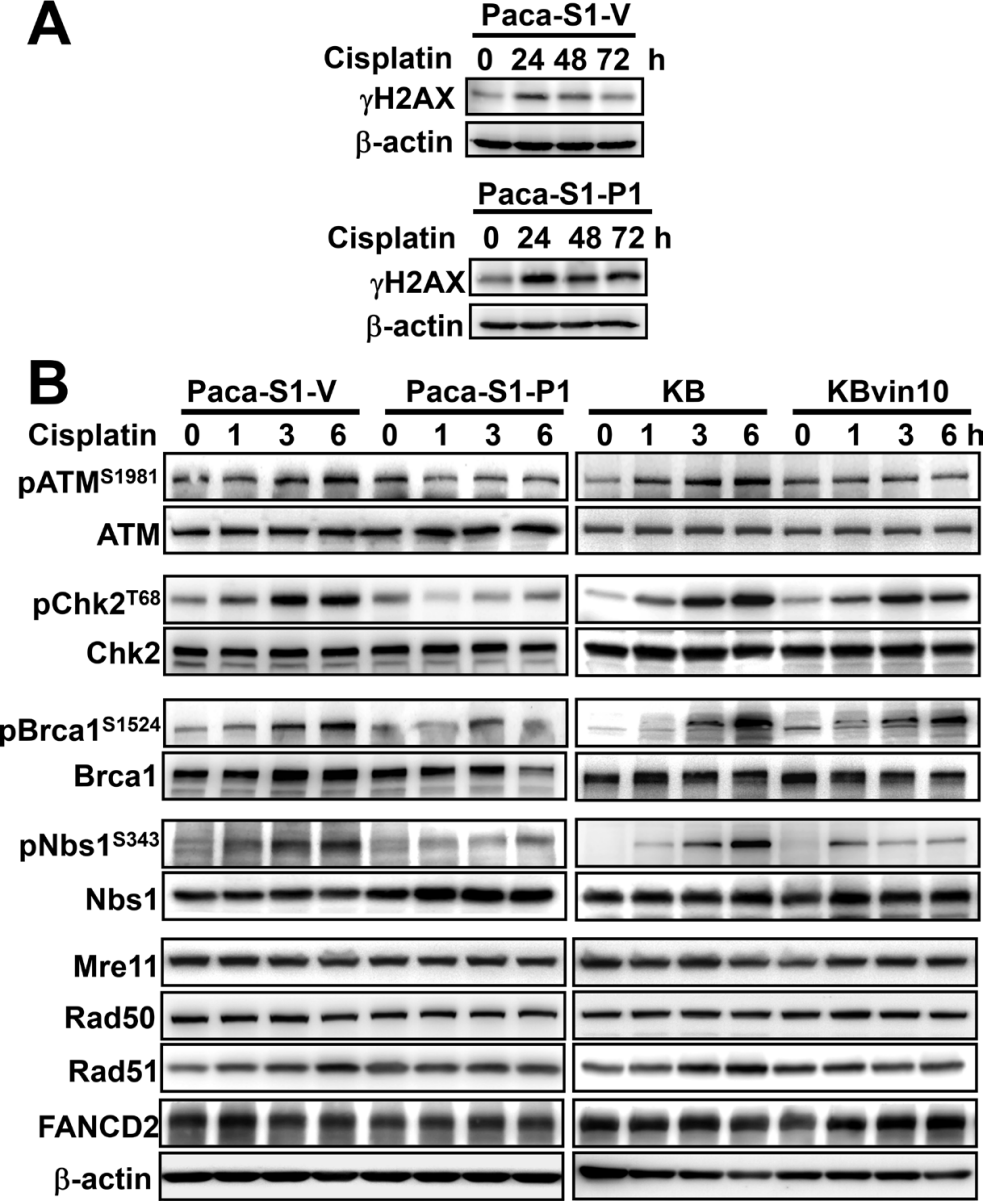
Reduced DNA damage response in cisplatin-treated P-gp overexpressing cells (**A**) Accumulated gH2AX in Paca-S1-P1 cells treated with cisplatin. Paca-S1-V and Paca-S1-P1 cells were treated with 50 μM cisplatin for 1 h, washed and cultured in drug-free medium for various time periods. The protein levels of γH2AX were determined by western blotting. (**B**) Attenuation of DNA repair protein activation by P-gp. Paca-S1-V and Paca-S1-P1 cells were treated with 50 μM cisplatin and KB and KBvin10 cells with 20 μM cisplatin for 1 h, washed, and incubated for 0, 1, 3 and 6 h. Afterward, several DDR related proteins with or without activated phosphorylation, such as ATM and pATM^S1981^, Chk2 and pChk2^T68^, Brca1 and pBrca1^S1524^, Nbs1 and pNbs1^S343^, and total proteins of Mre11, Rad50, Rad51, and FANCD2 were analyzed by western blotting. β-actin was included as a loading control.

Since we have shown increased susceptibility to DNA crosslinking agents in P-gp overexpressing cells, we then adopted phosphorylated histone H2AX (γH2AX) as DNA damage marker to further confirm the interference of DNA repair in P-gp overexpressing cells. As shown in Figure 3A, γH2AX was significantly increased at 24 h and gradually declined at 48 and 72 h in Paca-S1-V, indicating that the damaged DNA was gradually repaired. However, the levels of γH2AX were constantly maintained in Paca-S1-P1 cells up to 72 h, implying no significant DNA repair in Paca-S1-P1 cells. Similar results were observed in Paca-S1-V and Paca-S1-P1 cells treated with BO-1922, which is a potent agent to induce DNA interstrand crosslinks [42] (Supplementary Figure 2). These results implicated that attenuated Src activation by overexpressed P-gp may interfere with DDR. As shown in Figure 3B, we did not observed the change of protein levels of several proteins involved in DDR, such as ATM, Chk2, Brca1, Nbs1, Mre11, Rad50, Rad51, and FANCD2, in cisplatin treated cells either with P-gp overexpression (Paca-S1-P1 and KBvin10 cells) or without (Paca-S1-V cells and KB cells). However, we found that in response to cisplatin treatment the phosphorylated ATM (pATM^S1981^), Chk2 (pChk2^T68^), Brca1 (pBrca1^S1524^), and Nbs1 (pNbs1^S343^) were increased in a time-dependent manner in Paca-S1-V cells and KB cells, respectively. In cisplatin-treated Paca-S1-P1 and KBvin10 cells, the levels of phosphorylation of these proteins were apparently less than that in Paca-S1-V cells and KB cells, respectively. These results supported that P-gp attenuated Src activation and subsequently prevented the activation of DDR signaling in cells treated with DNA damaging agents. Consequently, P-gp overexpressing cells were more susceptible to DNA cross-linking agents compared to their parental cells.

### P-glycoprotein interacts with Src, Cbp, and Csk

**Figure 4:**
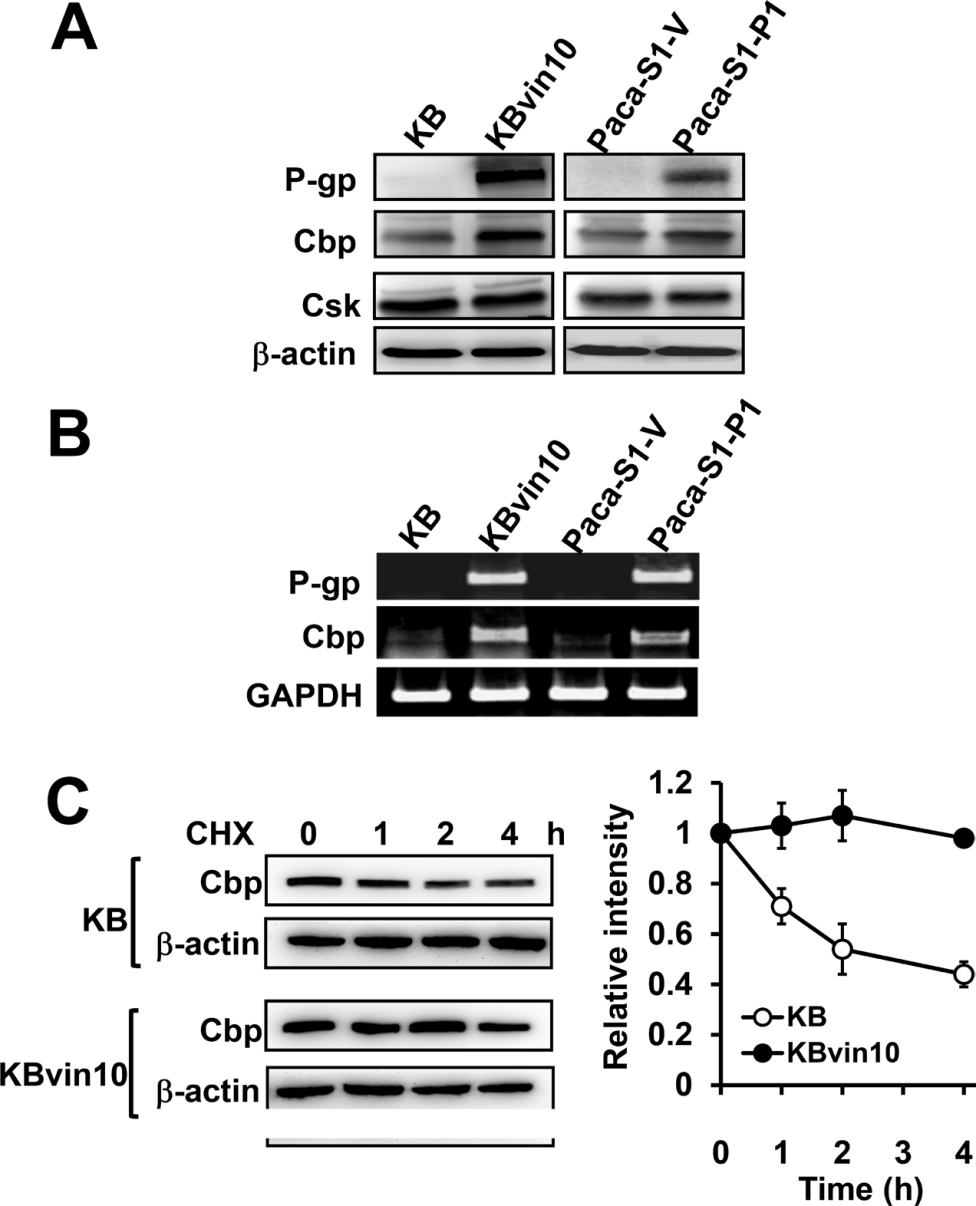
Increased Cbp expression and stability in P-gp overexpressing cells (**A** and **B**) Increased Cbp protein (A) and mRNA (B) in P-gp-overexpressing cells. The protein levels of P-gp, Cbp and Csk and the mRNA levels of P-gp and Cbp in KB, KBvin10, Paca-S1-V and Paca-S1-P1 cells were analyzed by western blotting and RT-PCR, respectivley. β-actin was included as a loading control in western blotting assay, while GAPDH expression served as an internal control of RT-PCR assay. (**C**) Increased Cbp stability in P-gp-overexpressing KBvin10 cells. KB and KBvin10 cells were incubated with cycloheximide (CHX) (20 μg/ml) for the indicated time periods. The protein levels of Cbp were determined by western blotting (left). β-actin was included as a loading control. The relative intensity of Cbp (right) was determined by imaging software. Bars are SD of three independent experiments.

Src is negatively regulated by Csk, which phosphorylates Src at Y527 [45]. While Csk was recruited to the membrane by the Src suppressor Cbp [40], P-gp was reported to recruit Cbp to the membrane [41]. We therefore investigated the role of Csk and Cbp in P-gp resulted in suppression of DNA damage induced Src activation. We first observed that P-gp overexpression did not affect Csk protein levels (Figure 4A). However, Cbp protein levels (Figure 4A) and mRNA levels (Figure 4B) were significantly enhanced in KBvin10 and Paca-S1-P1 cells compared to their parental KB and Paca-S1-V cells, respectively. We further observed a slow degradation of Cbp in KBvin10 cells compared with KB cells after treatment with cycloheximide (CHX) (Figure 4C). The estimated half-life of Cbp in KB cells was approximately 2 h, whereas no apparent Cbp degradation was observed in KBvin10 cells within 4 h. These results implied that P-gp not only enhances the expression of Cbp but also prolongs the stability of Cbp.

**Figure 5:**
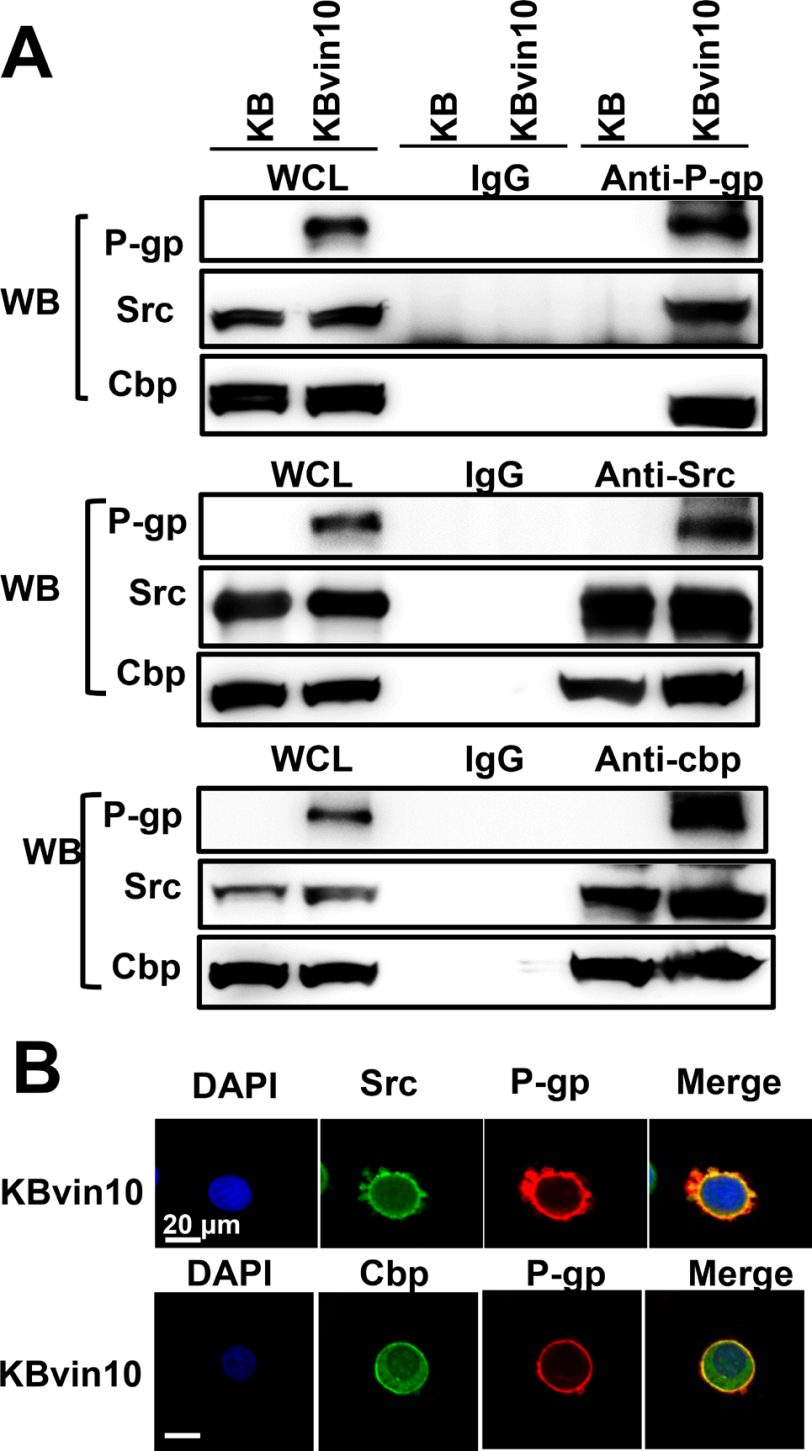
Interaction among P-gp, Src, and Cbp (**A**) Co-immunoprecipitation of P-gp, Src, and Cbp. Cell lysates from KB and KBvin10 cells were immunoprecipitated with antibodies against P-gp, Src or Cbp. The immuno-complexes were reciprocally subjected to western blotting using P-gp, Src, and Cbp antibodies, respectively. (**B**) Co-localization of P-gp, Src, and Cbp in KBvin10 cells. Logarithmically growing cells were fixed and stained with immunofluorescent antibodies as described in MATERIALS AND METHODS. The images were acquired by confocal microscopy.

**Figure 6:**
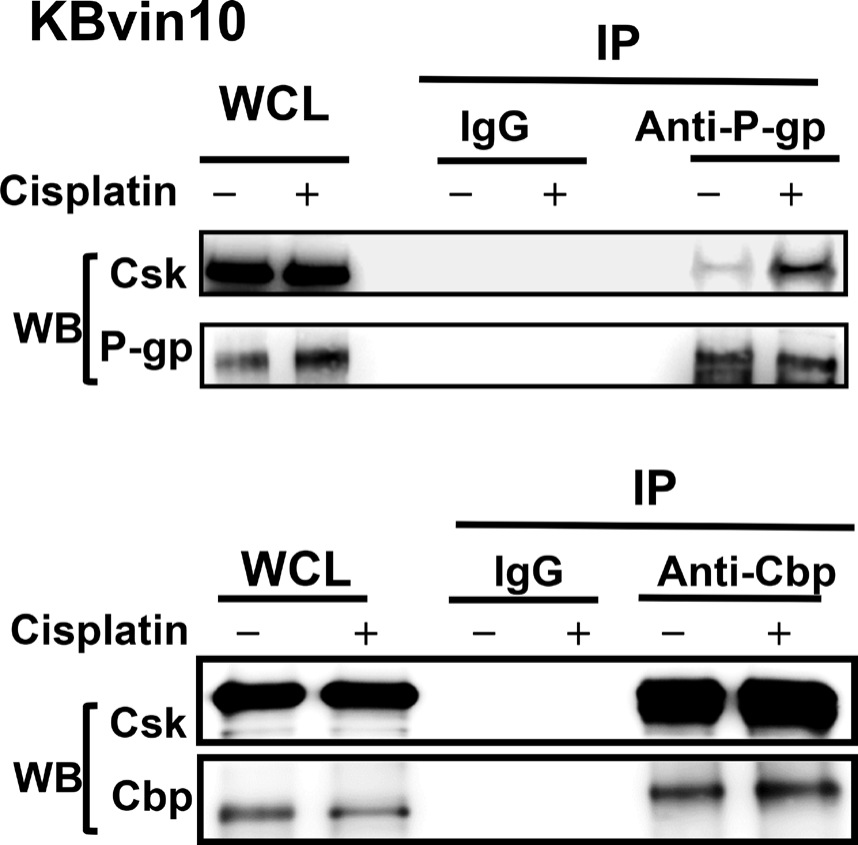
Association of P-gp with Csk in cisplatin-treated KBvin10 cells KBvin10 cells were treated with or without cisplatin at 20 μM for 1 h, washed, and then incubated with fresh medium for 2 h. Cell lysates were immunoprecipitated with antibodies against P-gp or Cbp and followed by western blotting with anti-P-gp, anti-Csk, or anti-Cbp antibodies, respectively. Normal mouse immunoglobulin (IgG) was included as a negative control and 1/10 of whole cell lysates (WCL) used for immunoprecipitation was included as loading control.

To explore the underlying mechanism through which P-gp attenuates Src activation, we examined the interaction between P-gp, Src, and Cbp by reciprocal immunoprecipitation assays using antibodies against P-gp, Src, and Cbp, respectively. As shown in Figure 5A, we confirmed the interaction between Src and Cbp. However, using antibody against one of these proteins, we observed that the other 2 proteins were co-precipiated in lysates of KBvin10 cells. These results revealed a direct interaction between P-gp, Cbp and Src in P-gp overexpressing KBvin10 The co-localization of P-gp and Src as well as P-gp and Cbp on the plasma membrane was further confirmed by immunofluorescence analysis in KBvin10 cells (Figure 5B). Similar results were found in Paca-S1 cells (Supplementary Figure 3). In addition, a large portion of Src and Cbp were localized in the cytosol. Intriguingly, the interaction of P-gp and Csk was limited in KBvin10 cells without exposure to DNA-damaging agents but significantly increased upon cisplatin treatment (Figure 6). However, the interaction of Cbp and Csk was not affected by cisplatin treatment. Accordingly, we may infer that upon cisplatin treatment P-gp enhances the recruitment of Csk from the cytosol to the cell membrane, where Csk phosphorylates Src at Y527 and attenuates Src activation.

### P-glycoprotein and Cbp silencing restores cisplatin-induced activation of Src at Y416 and DNA repair capacity in MDR cells

**Figure 7:**
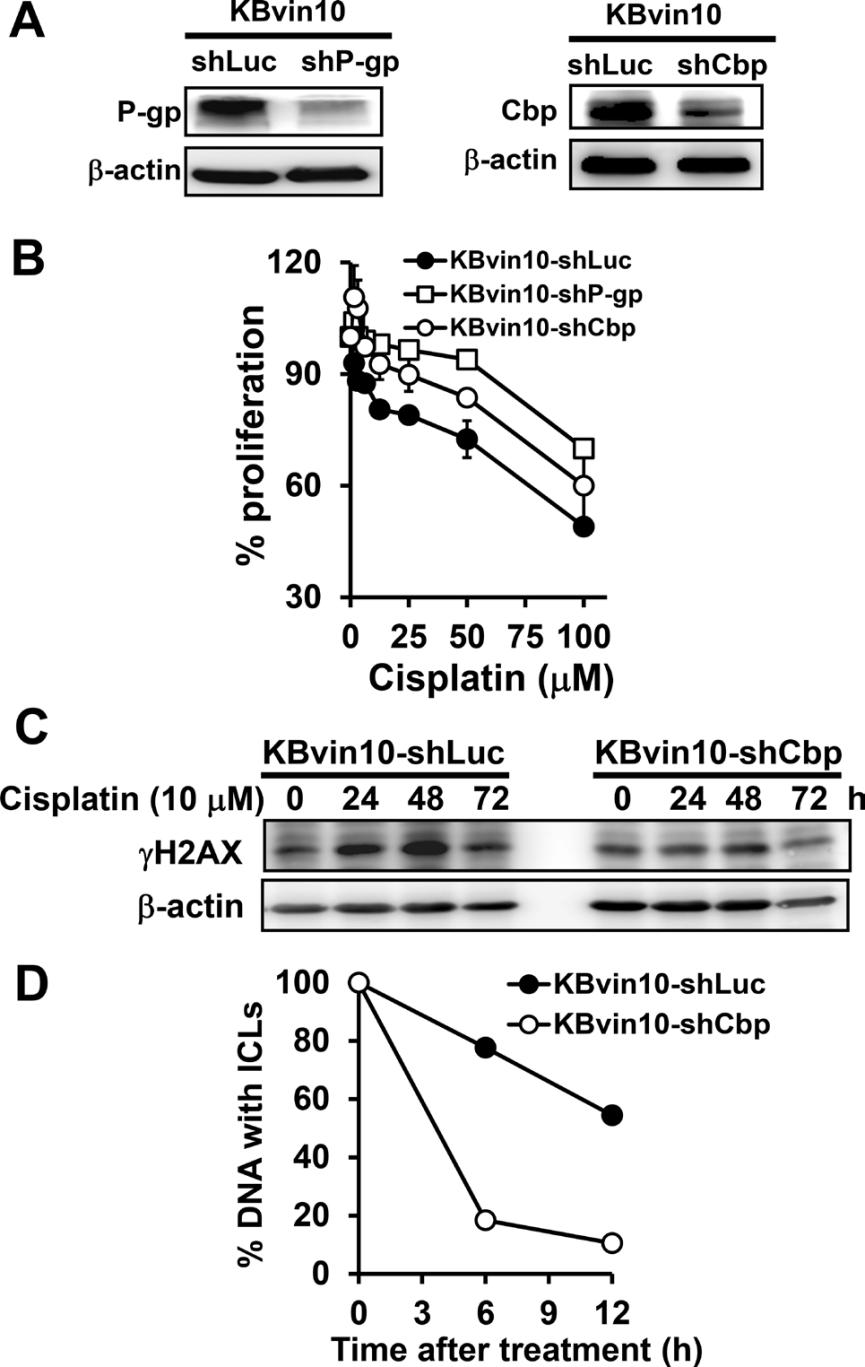
Increased resistance to DNA-damaging agents by P-gp or Cbp silencing (**A**) Reduced P-gp or Cbp by shP-gp or shCbp, respectively. KBvin10 cells were infected with lentiviral shLuc, shP-gp, or shCbp for 24 h, washed, and cultured in medium containing 4 μg/ml puromycin. Four days after selection, P-gp and Cbp proteins in KBvin10-shLuc, -shP-gp, or -shCbp cells were analyzed by western blotting. β-actin was included as a loading control. (**B**) Increased survival rate in P-gp- or Cbp-silenced cells treated with DNA-damaging agents. KBvin10-shLuc, -shP-gp, or -shCbp cells were treated with various concentrations of cisplatin for 1 h, washed with PBS, and incubated for 72 h. The cell proliferation was determined using Presto-Blue agents as described previously. Bars are SD of three independent experiments. (**C**) Decreased accumulation of gH2AX in cisplatin-treated KBvin10-shCbp cells. KBvin10-shLuc and -shCbp cells were treated with 10 μM cisplatin for 1 h, washed, and changed to drug-free medium for the indicated time. γH2AX levels were analyzed by western blotting. (**D**) Facilitating the repair of cisplatin-induced DNA ICLs in Cbp silencing cells. KBvin10-shLuc and KBvin10-shCbp cells were treated with 10 μM cisplatin for 1 h. Cisplatin-induced DNA ICLs were determined by a modified comet assay after incubation in drug-free medium for different time periods. Data are average of 2 independent experiments.

To further reveal the roles of P-gp and Cbp in the suppression of cisplatin-induced Src activation, we established P-gp- and Cbp silenced KBvin10 cells by infecting the cells with P-gp or Cbp lentiviral shRNA, respectively. KBvin10-shLuc cells infected with lentiviral luciferase shRNA were used as a control. Reduced P-gp and Cbp protein levels in KBvin10-shP-gp and KBvin10-shCbp compared to KBvin10-shLuc cells was demonstrated by western blotting analysis (Figure 7A). We also confirmed increased resistance to cisplatin in P-gp or Cbp silenced KBvin10 cells (Figure 7B). As described above, we inferred that P-gp restrained Cbp turnover, resulting in its accumulation and hence suppression of DNA damage-induced Src activation. We further demonstrated that the DNA damage marker γH2AX accumulated in KBvin10-shLuc cells treated with cisplatin up to 48 h and then declined (Figure 7C). However, no significant increase in γH2AX was observed in cisplatin-treated KBvin10-shCbp cells (Figure 7C), implying that cisplatin-induced DNA damages were rapidly rescued and repaired in KBvin10-shCbp cells compared with KBvin10-shLuc cells. To confirm this finding, we adopted a modified comet assay to compare the repair activity of DNA ICLs in KBvin10-shLuc and KBvin10-shCbp cells. As shown in Figure 7D, Cbp silencing accelerated the repair of DNA ICLs.

**Figure 8:**
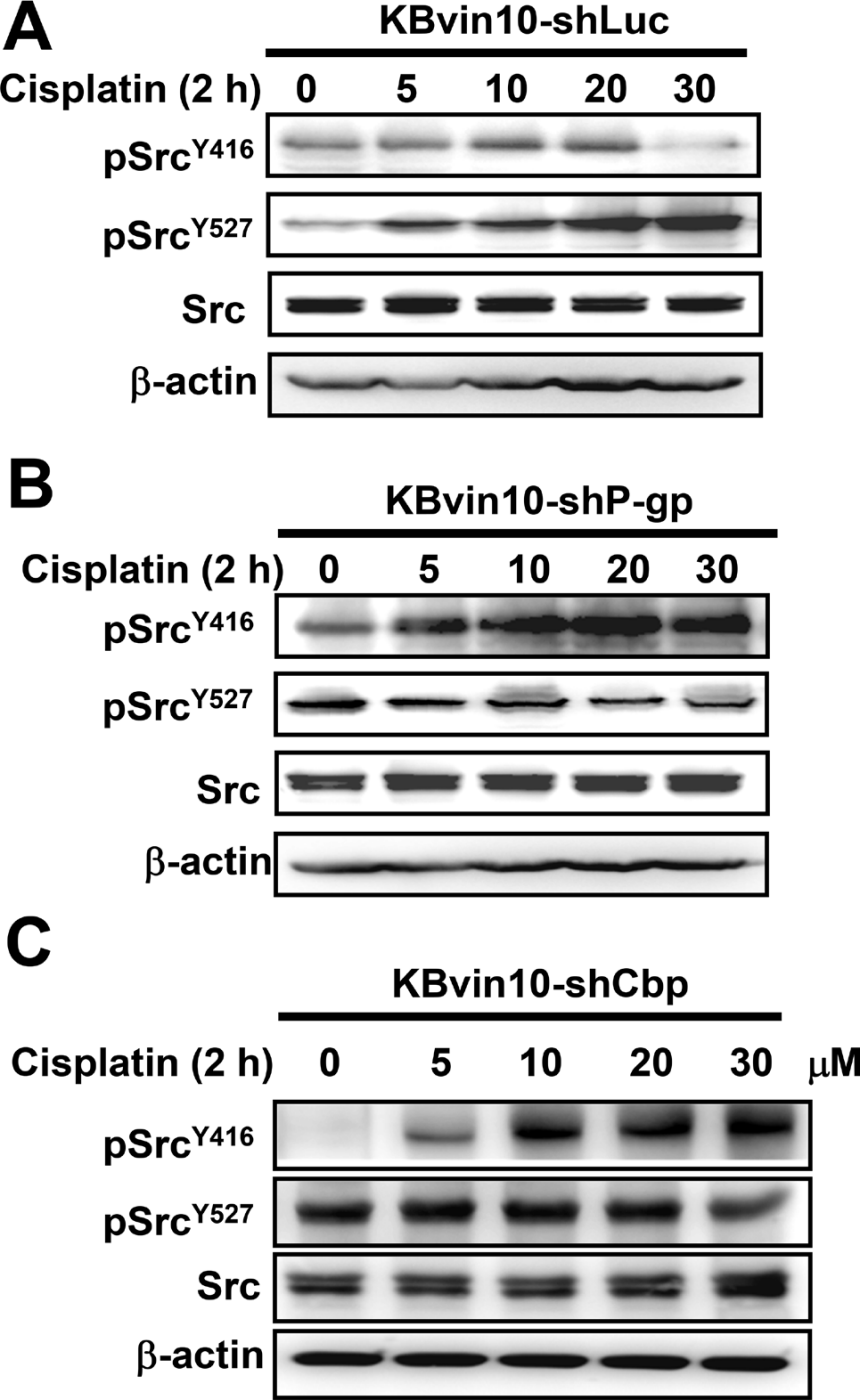
Enhanced Src activation in P-gp- or Cbp-silenced KBvin10 cells by cisplatin KBvin10-shLuc (**A**), -shP-pg (**B**), and -shCbp (**C**) cells were treated with cisplatin at various concentrations for 1 h, washed, and incubated with fresh medium for 2 h. The levels of total Src, pSrc^Y416^, and pSrc^Y527^ were determined by western blotting. β-actin was included as a loading control.

Moreover, the effects of cisplatin treatment on Src activation in KBvin10-shLuc, KBvin10-shP-gp, and KBvin10-shCbp cells were examined by determining the levels of pSrc^Y416^ and pSrc^Y527^. Similar to the parental KBvin10 cells, no significant increase of pSrc^Y416^ was observed in the cisplatin-treated vector control KBvin10-shLuc cells (Figure 8A). Alternatively, pSrc^Y527^ was significantly increased in cisplatin-treated control KBvin10-shLuc (Figure 8A). However, activated pSrc^Y416^ was significantly increased by cisplatin in both KBvin10-shP-gp cells (Figure 8B) and Cbp-silenced KBvin10-shCbp cells (Figure 8C). Consistently, the protein levels of inactivated pSrc^Y527^ were reduced in cisplatin treated KBvin10-shP-gp cells (Figure 8B) and KBvin10-shCbp cells (Figure 8C). These results further support that P-gp and Cbp may serve as negative regulators of Src. Their overexpression in MDR cells was likely to sensitize the MDR cells to DNA cross-linking agents.

### Ectopic expression of Cbp inhibits cisplatin-induced activation of Src in KB cells

**Figure 9:**
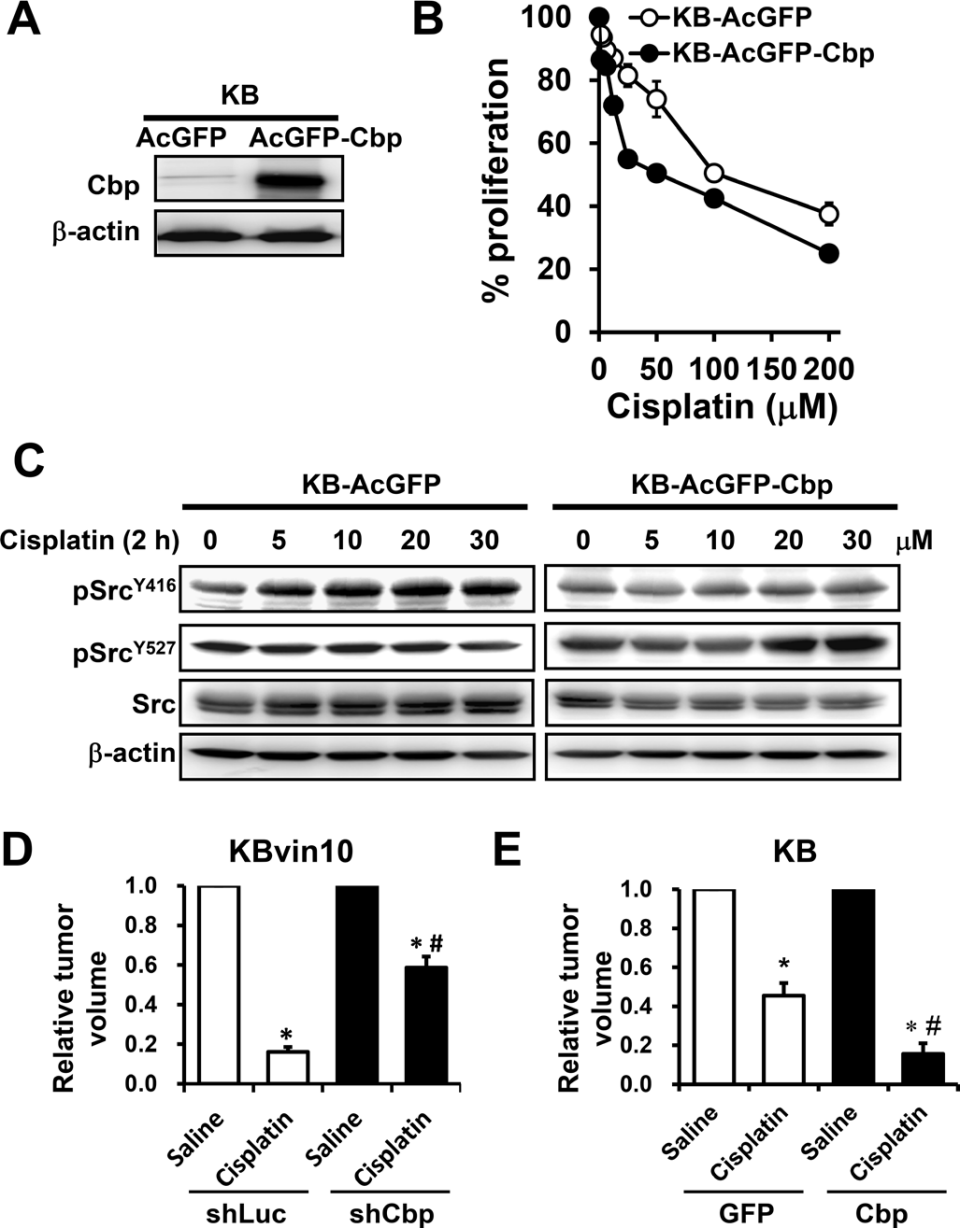
Attenuated Src activation by cisplatin in Cbp-overexpressing cells (**A**) Enhanced Cbp expression in KB cells transfected with the Cbp expression vector. Stable cell lines were cloned and maintained in medium containing G418. The protein levels of Cbp in KB-AcGFP cells and KB-AcGFP-Cbp cells were analyzed by western blotting. (**B**) Increased sensitivity to DNA-damaging agents in Cbp-overexpressing cells. KB, KB-AcGFP and KB-AcGFP-Cbp cells were treated with cisplatin for 1 h, washed and then incubated with fresh medium for 72 h. The cell proliferation was determined using Presto-Blue. (**C**) Attenuation of Src activation by Cbp. KB-AcGFP and KB-AcGFP-Cbp cells were treated with cisplatin at various concentrations for 1 h, washed, and incubated with fresh medium for 2 h. The levels of total Src, pSrc^Y416^, and pSrc^Y527^ were determined by western blotting. β-actin was included as a loading control. (**D** and **E**) Increased anti-tumor activity of cisplatin by enhanced Cbp expression. Athymic nude mice bearing cancer xenografts, derived from KBvin10-shLuc and KBvin10-shCbp cells (D) and from KB-GFP and KB-Cbp cells (**E**) were intratumorally treated with one dose of saline or cisplatin (4 mg/kg body weight) when the tumor reached a volume of 100 mm^3^ (6 mice/each group). Ten days after treatment, the tumor volumes were determined. Each bar represents the mean ± S.E of 6 mice from 2 independent experiments. Statistical significance was determined by one-way ANOVAs. *p < 0.05, saline vs. cisplatin; ^#^*p* < 0.05, KBvin10-shLuc *vs* KBvin10-shCbp or KB-GFP *vs* KB-Cbp cells treated with cisplatin.

To further verify the inhibitory role of Cbp in DNA-damaging agent-induced Src activation, we ectopically expressed Cbp in KB cells and established stable clones (KB-AcGFP-Cbp) (Figure 9A). We observed that KB-AcGFP-Cbp cells were more susceptible to cisplatin than KB and control KB-AcGFP cells (Figure 9B). Furthermore, cisplatin treatment failed to induce pSrc^Y416^ but significantly increased pSrc^Y527^ in KB-AcGFP-Cbp cells (Figure 9C), supporting the inhibitory role of Cbp in cisplatin-induced Src activation. We further performed an *in vivo* assay to evaluate the therapeutic activity of cisplatin against KBvin10 cells with differential expression of Cbp. As shown in Figure 9D, the KBvin10-shLuc and KBvin10-shCbp xenografts were treated *in situ* with normal saline or cisplatin at 4 mg/kg. At day 10, cisplatin suppressed the growth of the KBvin10-shLuc and KBvin10-shCbp xenografts by 80.5% and 41.1%, respectively, suggesting that silencing of Cbp resulted in increased cisplatin resistance. Similarly, cisplatin treatment reduced the tumor growth of KB-GFP and KB-Cbp xenografts by 54.6% and 80.7%, respectively (Figure 9E), confirming that enhanced expression of Cbp sensitizes the cells to cisplatin. Together, these results indicate that P-gp interacts with Cbp and Src to recruit Csk to attenuate Src activation upon DNA injury, a crucial signal for activation of the DNA repair machinery.

## DISCUSSION

Although P-gp acts as an efflux pump responsible for preventing the delivery of various chemotherapeutic agents to cancer cells, several reports have demonstrated that increased P-gp levels enhance the sensitivity of MDR cancer cells to various DNA-damaging agents, such as cisplatin, bis-chloroethylnitrosourea, and gemcitabine [14, 15, 46]. Several possible explanations have been reported to account for the toxicity of P-gp-potentiated compounds, including generation of reactive oxygen species through ATP hydrolysis, altered drug metabolism, and membrane perturbation [47, 48]. We have previously shown that overexpressed P-gp in MDR cells attenuates the Src-activated repair activity by DNA ICLs and contributes to the susceptibility of MDR cells to DNA ICL agents [16]. In this report, we revealed mechanistic insights regarding how P-gp interferes with Src activation and DNA repair activity. Our results first demonstrated that the expression levels of P-gp were inversely associated with Src activation and repair of cisplatin-induced DNA ICLs. Second, we noticed that P-gp overexpression attenuated the DDR upon treatment with DNA damaging agents. Third, we observed significantly increased expression of Cbp in P-gp-overexpressing cells, in which P-gp interacts with Cbp and prolongs its stability. Fourth, upon cisplatin treatment, we revealed an interaction between P-gp and Csk in KBvin10 cells in which Cbp levels were enhanced. Fifth, the repair of cisplatin-induced ICLs and Src activation were suppressed by ectopically expressed Cbp, whereas Cbp silencing enhanced cisplatin-induced Src activation and increased the resistance of MDR cells to DNA ICL agents. Together, our present results revealed that overexpressed P-gp in MDR cells mediates through a Cbp-dependent mechanism to attenuate and Src activation and DNA repair activity.

Src and other Src family kinases are overexpressed and participate in numerous signaling pathways in various human cancers [49]. Activated Src has also been shown to cause cancer cells to be resistant to various chemotherapeutic agents and targeted therapeutics [50, 51]. Actually, numerous reports have demonstrated that Src activation is involved in drug resistance by enhancing DNA repair [25–27]. Our present results showing that cisplatin treatment significantly increased the levels of pSrc^Y416^ (activated Src) as well as pEGFR^Y845^ in KB cells but not in P-gp overexpressed KBvin10 cells. The phosphorylation of EGFR at Y845 was mediated by activated Src [43] and involved in regulation of DNA-repair [26]. Furthermore, we found that overexpressed P-gp, either in acquired MDR cells or in ectopically expressed cells, interfered with cisplatin-induced phosphorylation of ATM, Chk2, Brca1 and Nbs1 that have been known as major regulators of DDR, which activate different DNA repair processes, including homologous recombination repair, non-homologous end joining repair [52]. These results supported that suppression of DNA-damaging agent-induced Src activation by P-gp is likely to inhibit DDR in MDR cells. This may be a reason to explain why MDR cells are more susceptible to cisplatin or other DNA ICL agents than the parental cells.

Uncovering the mechanisms of Src regulation and activation will probably provide new clues to guide cancer treatment [29, 53]. In general, Src is activated via autophosphorylation of the tyrosine residue Y416 in its SH1 kinase domain and inactivated by phosphorylation of the negative regulator Y527 at its carboxy-terminal domain by Csk [49, 54]. Our current results revealed that the enhanced expression of P-gp reduces cisplatin-induced active pSrc^Y416^ levels but significantly increases inactive pSrc^Y527^ levels, implying that P-gp may increase the access of cytosolic Csk to membrane-anchored Src. However, overexpressed P-gp does not change Csk protein levels. Csk, existing in the cytoplasm for lacking a fatty acetylation site [55], must be recruited to the plasma membrane to exert its function. We further found that Cbp, a Csk binding protein that efficiently recruits cytosolic Csk to the plasma membrane was significantly increased in P-gp overexpressing cells. Therefore, we infer that overexpression of P-gp and Cbp may facilitate recruitment of Csk to the membrane and result in phosphorylation of Src at Y527 upon treatment with DNA-damaging agents.

Src is mainly anchored to membranes *via* its N-terminal myristolyation and adaptor protein [18, 36]. Numerous studies have shown that P-gp and Src are associated with lipid rafts [40, 56], where lipid raft modulation inhibits P-gp and limits the transforming potential of Src [57, 58]. Because we observed partial colocalization of P-gp, Cbp and Src on the plasma membrane, P-gp may upregulate Cbp to interfere with Src activation in cells treated with DNA-damaging agents. In addition to recruiting Csk to lipid raft-associated Src kinase, Cbp can also potentiate Csk enzymatic activity [59]. Numerous reports have shown that the Cbp-Csk-Src pathway is involved in the regulation of Src signaling in response to diverse stimuli [60–62]. Our results indicated that Cbp was closely associated with P-gp overexpression in either acquired MDR cells, KBvin10, or ectopically expressed cells, Paca-S1-P1. We may further infer that P-gp is involved in facilitating the negative regulatory Cbp-Csk-Src pathway, hence sensitizing MDR cells to certain DNA-damaging agents, such as cisplatin.

The mechanism of Cbp upregulation, particularly how P-gp enhances Cbp expression, remains elusive. P-gp is a lipid translocase that induces the translocation of sphingomyelin and glucosylceramide across the plasma membrane [56]. Recent studies have shown that P-gp interferes with membrane lipid organization and facilitates the recruitment of Cbp [41]. Our present study demonstrated that enhanced Cbp in P-gp overexpression cells is likely because of increased transcription and protein stability. However, the interaction domains between P-gp and Cbp warrant further investigation.

Our study supports our previous finding that P-gp may play a special role in the inhibition of Src activation in cells exposed to DNA-damaging agents [16]. We further identified a critical role of Cbp in regulating P-gp-mediated Src activation and in sensitizing cancer cells to ICL agents. We also demonstrated that enhanced expression of Cbp could sensitize the xenografts to cisplatin in mice. These findings suggest that P-gp overexpressing cells might interfere with Cbp-Csk-Src cascade and DDR and consequently enhance the sensitivity to DNA damaging agents, such as cisplatin or other DNA ICL agents. Therefore, DNA ICL agents might be potential therapeutic drugs against P-gp-overexpressing MDR cells, and Cbp mimics could serve as a potential agent for anticancer therapy.

## MATERIALS AND METHODS

### Chemicals and reagents

Vincristine, vinblastine, etoposide, paclitaxel (Taxol), cisplatin, carboplatin and melphalan were purchased from Sigma-Aldrich (St Louis, MO, USA), and doxorubicin from MP Biomedicals (Santa Ana, CA, USA). Cell culture chemicals were obtained from Gibco (Grand Island, NY, USA), and other reagents from Merck (Darmstadt, Germany). BO-1922 was synthesized according to our published procedure [42]. Cisplatin was freshly prepared by dissolving in dimethyl sulfoxide, immediately diluted with medium, and administrated into the culture dishes or wells.

### Cell lines and cell culture

KB and KBvin10 cells, obtained from Dr. J. Y. Chang (National Health Research Institutes, Taiwan), were cultured as previously described [16]. KBvin10 cells overexpressing P-gp and vincristine resistance were derived from KB cells, which were originally thought to be an oral epidermal carcinoma cell line but with HeLa cell characteristics. Paca-S1 cells, derived from a pancreatic carcinoma Mia-Paca-2 xenograft by Dr. H. C. Wu (Institute of Cellular and Organismic Biology, Academia Sinica, Taiwan), were cultured in DMEM supplemented with 10% FBS and antibiotics.

### Cytotoxicity assay

The cytotoxicity of various cell lines to various compounds was assessed using Presto Blue^®^ (Life Technologies Co.) according to the manufacturer's instructions. Briefly, 3,000-5,000 cells were seeded in each well of 96-well plates and incubated at 37°C overnight prior to drug treatment. In general, the cells were treated with various concentrations of drugs for 72 h. At the end of treatment, an aliquot of Presto-Blue solution (at 1/10 volume) was added to each well, and the plates were further incubated at 37°C for 3 h. Cell proliferation was assessed by determining the absorbance at 570/600 nm using a microplate spectrophotometer, and the IC_50_ values of each compound were calculated from the dose-effect relationships at six or seven concentrations using CompuSyn software (version 1.0.1; CompuSyn, Inc.) by Chou and Martin based on the median-effect principle and plot [63].

### Western blot analysis

Western blotting was performed as previously described [64]. Equal amounts of cell lysates or immunoprecipitates were resolved by sodium dodecyl sulfate-polyacrylamide gel electrophoresis and subjected to western blotting using the following antibodies. Primary antibodies against P-gp, Csk and FANCD2 were obtained from Santa Cruz Biotechnology (Santa Cruz, CA, USA), Src, pSrc^Y416^, pSrc^Y527^, ATM, pATM^S1981^, Chk2, pChk2^T68^, Brca1, pBrac1^S1524^, Nbs1, pNbs1^S343^, Mre11 and Rad50 from Cell Signaling Technology (Danvers, MA, USA), Rad51 from GeneTex (Irvine, CA, USA), γH2AX from Calbiochem (San Diego, CA, USA), and Cbp and β-actin from Abcam (Cambridge, MA, USA). The secondary horseradish peroxidase-conjugated antibody was purchased from Abcam (Cambridge, MA, USA). The specific protein bands were visualized by chemiluminescence using the SuperSignal West Pico chemiluminescence reagent (Pierce, Rockford, IL).

### Ectopic expression

P-gp and Cbp were inserted into the pCMV6-ENTRY and pCMV6-AC-GFP (OriGene Technologies, Rockville, MD, USA) expression vectors, respectively. The constructed plasmids were transfected into cells by Lipofectamine 2000 according to the manufacturer's instructions. Stable transfectants were selected by culturing the cells with 0.5 g/ml G418 (Gibco) for 2 weeks. Paca-S1 cells stably expressing P-gp were maintained in the presence of 10 nM vincristine.

### Gene silencing by shRNA

The lentiviruses containing the expression vectors of shRNAs of P-gp (pLKO.1-shP-gp) and Cbp (pLKO.1-shCbp) were obtained from the National RNAi Core Facility located at Academia Sinica, Taipei, Taiwan. The sequences of the P-gp and Cbp shRNAs were 5′-GCTCATCGTTTGTCTACAGTT-3′ and 5′-TGATCTCTATGCTACTGTTAA-3′, respectively. The lentiviral expression vector pLKO.1-shLuc, containing 5′-GCGGTTGCCAAGAGGTTCCAT-3′ specific for luciferase, was used as a vector control. To silence the expression of P-gp or Cbp, KBvin10 cells were infected with lentivirus containing the specific shRNA. These cell lines were maintained in the presence of 4 μg/ml puromycin.

### Reverse transcription polymerase chain reaction (PCR)

Total RNA was extracted by using Trireagent (Sigma). The total RNAs were subjected to reverse transcription with SuperScript III (Invitrogen) for RNA synthesis. PCR was performed with the following pairs of specific primers: 5′-TCCGGTTTGGAGCCTACTTGG-3′ and 5′-AGGCATGTATGTTGGCCTCC-3′ for P-gp, 5′-TGGGGACATTCTTTCAGAGG-3′ and 5′-GGTGGAC TCCGGAACTGTAA-3′ for Cbp, 5′-CGAGATCCCTCC AAAATCAA-3′ and 5′-TGCTGTAGCCAAATTCGTTG-3′ for the glyceraldehyde-3-phosphate dehydrogenase gene (GAPDH). The PCR products were examined by electrophoresis on 2% agarose gels, stained with ethidium bromide, and visualized under UV light.

### Immunoprecipitation

Cell were lysed in ice-cold lysis buffer (50 mM Tris-HCl at pH 7.5, 150 mM NaCl, 1 mM EDTA pH 8, 1% NP-40, and 0.25% deoxycholate containing a protease inhibitor cocktail set (Calbiochem) and 1 mM each of phenylmethylsulfonyl fluoride and sodium orthovanadate) and pre-cleared by incubating with protein G-Sepharose beads for 30 min. Pre-cleared lysates were incubated with the indicated primary antibody or isotype-specific IgG as a control overnight at 4°C, followed by incubation with protein G-Sepharose beads (Millipore, Billerica, MA, USA) for 2 h at 4°C. Immunocomplex beads were washed 3 times with PBS, immediately boiled in 2× sample buffer and then processed for electrophoresis and western blotting.

### Immunofluorescence staining

To visualize the intracellular location of P-gp, Src or Cbp, KBvin10 or Paca-S1 cells were cultured on slides, washed with cold PBS and fixed with 100% ice-cold methanol for 30 min. Slides were then washed twice with PBS, permeabilized with PBS containing 0.2% Triton X-100 for 10 min and incubated overnight with appropriate antibody at 4°C, followed by Alexa Fluor 488-conjugated secondary goat anti rabbit IgG antibodies or Alexa Fluor 555-conjugated secondary goat anti mouse IgG antibodies (Molecular Probes, Eugene, OR, USA). The nuclei were counterstained with 4′,6-diamidino-2-phenylindole (DAPI) as previously described [65]. After mounting with 50% glycerol in PBS, images were acquired with a laser scanning confocal microscope (LSM 700, Carl Zeiss Microlmaging Inc., Thornwood, NY, USA) and AxioVision software.

### Modified comet assay

The repair of intercellular DNA cross-linking in cells was analyzed using the modified comet assay as previously described [66]. Briefly, cells were treated with cisplatin for 1 h at 37°C, followed by washing with PBS and incubating in fresh medium for 24 h. At the end of the incubation, the cells were X-ray irradiated at a dose of 20 Gy and immediately subjected to single-cell alkaline gel electrophoresis. The percentage of ICLs in cells was estimated by the percentage decrease in the tail moment of cells irradiated with 20 Gy alone.

### Mouse xenografts

Mouse xenografts were used to analyze the effects of cisplatin on the growth of cells expressing different levels of Cbp. The use of animals followed the guidelines approved by the Institutional Animal Care and Utilization Committee of the Academia Sinica (Taipei, Taiwan). Six-week-old male BALB/c nude mice, obtained from the National Laboratory Animal Center (Taipei, Taiwan), were housed in a specific pathogen-free environment under controlled light and humidity conditions as previously described [67]. To generate the xenografts, aliquots of approximately 5 × 10^6^ KB-GFP or KB-Cbp cells or 1 × 10^7^ KBvin10-shLuc or KBvin10-shCbp cells in 100 μl of PBS were inoculated subcutaneously into the flank region. When the average tumor size reached approximately 100 mm^3^, the mice were randomly divided into two groups (3 for each group) and intratumorally injected once with vehicle (saline) or cisplatin (dissolved in saline and given at 4 mg/kg body weight). Tumor volumes (mm^3^) were measured using calipers and calculated according to the formula: tumor volume = (length × width^2^)/2.

## SUPPLEMENTARY MATERIALS FIGURES AND TABLES



## Abbreviations

MDR: Multidrug resistance
ABC: ATP-binding cassette
P-gp: P-glycoprotein
ICL: interstrand cross-linking
DDR: DNA damage response
Csk: C-terminal Src kinase
Cbp: Csk-binding protein
γH2AX: phosphorylated histone H2AX
CHX: cycloheximide
PCR: polymerase chain reaction
