# Supplementary material for: P-glycoprotein attenuates DNA repair activity in multidrug-resistant cells by acting through the Cbp-Csk-Src cascade
Source: Oncotarget. 2017 Feb 3;8(28):45072–87. doi: 10.18632/oncotarget.15065 (PMC5542168; doi:10.18632/oncotarget.15065)
Supplement: Supplementary file 1 [file oncotarget-08-45072-s001.pdf]

P-glycoprotein attenuates DNA repair activity in multidrug-resistant cells by acting through the Cbp-Csk-Src cascade

Supplementary Materials

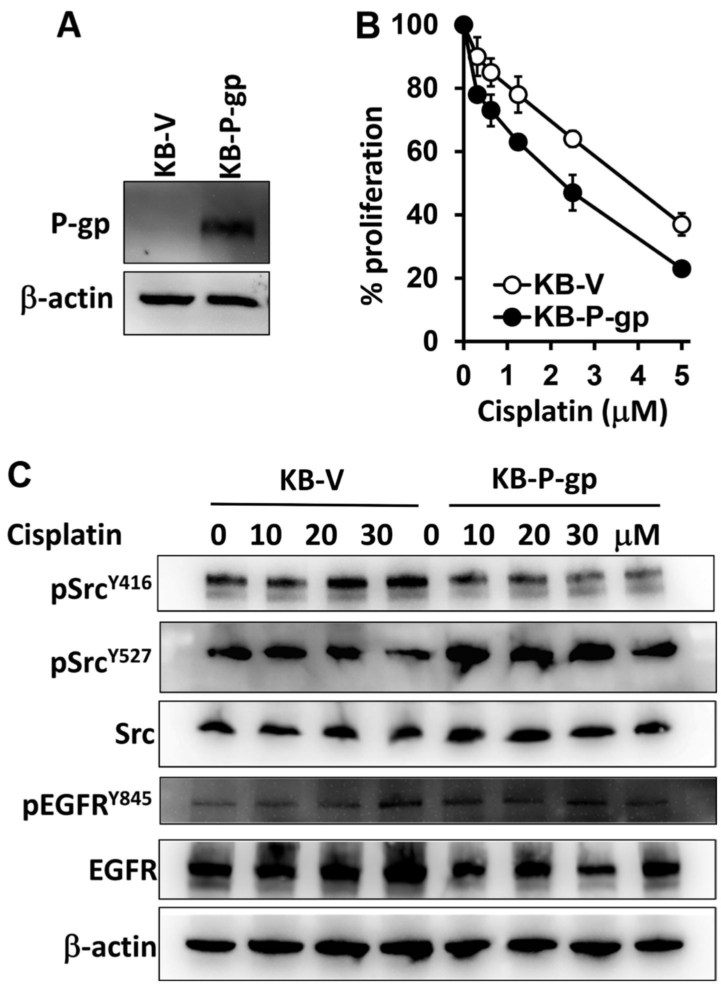

Supplementary Figure 1: Increased susceptibility to cisplatin by transient expression of P-gp in KB cells. (**A**) Increased expression of P-gp by transfection of P-gp expression vector into KB cells. The P-gp protein levels in KB-V and KB-P-gp cells were determined by western blotting. (**B**) Increasing cytotoxic effects of cisplatin to KB-P-gp cells. KB-V and KB-P-gp were treated with various concentrations of cisplatin for 72 h. The cell proliferation was analyzed using Presto-Blue. Bars are SD of three independent experiments. (**C**) No significant Src activation in KB-P-gp cells treated with cisplatin. KB-V or KB-P-gp cells were treated with various concentrations of cisplatin for 1 h, washed with PBS, and incubated with fresh medium for 2 h. The levels of Src, $pSrc^{Y416}$ (active) and $pSrc^{Y527}$ (inactive), EGFR and $pEGFR^{Y845}$ were determined by western blotting. β-actin was included as a loading control.

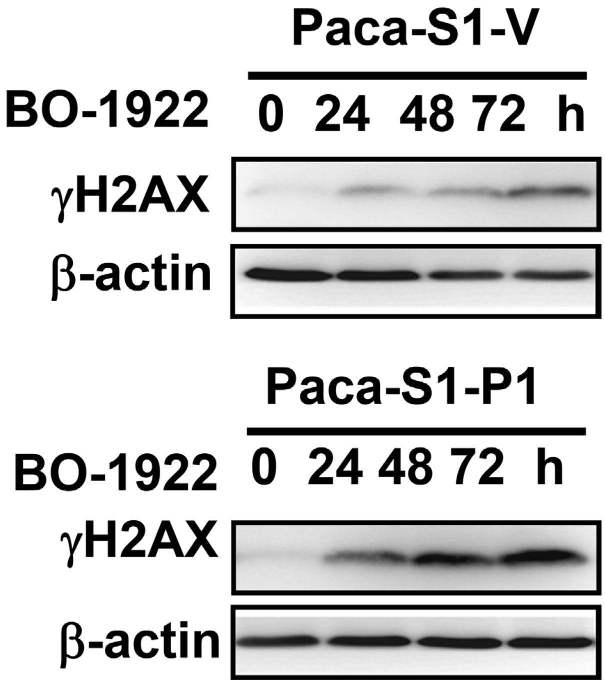

Supplementary Figure 2: Enhanced γH2AX accumulation in cisplatin-treated P-gp overexpressing cells. Paca-S1-V and Paca-S1-P1 cells were treated with 5 μM BO-1922 for 1 h, washed and cultured in drug-free medium for various time periods. The protein levels of γH2AX were determined by western blotting.

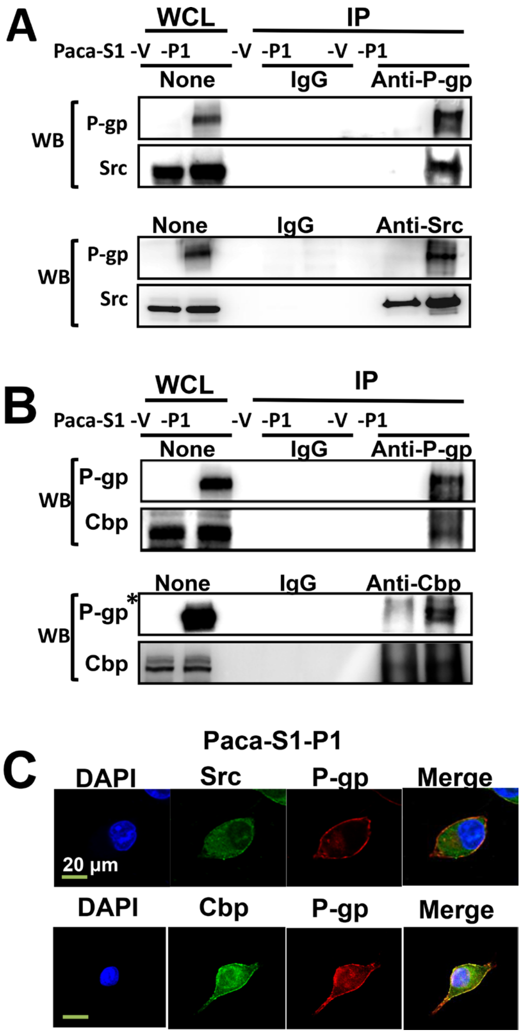

Supplementary Figure 3: Interaction among P-gp, Src, and Cbp in Paca-S1-P1 cells. (**A**) Co-immunoprecipitation of P-gp and Src. Cell lysates from Paca-S1-V and Paca-S1-P1 cells were immunoprecipitated with antibodies against Src or P-gp. The immunocomplexes were reciprocally subjected to western blotting using P-gp and Src antibodies, respectively. (**B**) Co-immunoprecipitation of P-gp and Cbp. As described in (A) cell lysates were immunoprecipitated with anti-P-gp or anti-Cbp and followed by western blotting with P-gp and Cbp antibodies, respectively. Normal mouse immunoglobulin (IgG) was included as a negative control, while 1/10 of whole cell lysates (WCL) used for immunoprecipitation was included as loading control. *, 1/20 of WCL was used to indicate the amount of P-gp. (**C**) Co-localization of P-gp, Src, and Cbp in Paca-S1-P1 cells. Logarithmically growing cells were fixed and stained with immunofluorescent antibodies as described in MATERIALS AND METHODS. The images were acquired by confocal microscopy.
